# Postoperative hypocalcemia: analysis of factors influencing early hypocalcemia development following thyroid surgery

**DOI:** 10.1186/s12893-019-0483-y

**Published:** 2019-04-24

**Authors:** Paolo Del Rio, Matteo Rossini, Chiara Montana Montana, Lorenzo Viani, Giuseppe Pedrazzi, Tommaso Loderer, Federico Cozzani

**Affiliations:** 1 Department of Medicine and Surgery, General Surgery Unit, University Hospital of Parma, Parma, Italy; 2 Department of Medicine and Surgery, University of Parma, Parma, Italy

**Keywords:** Hypocalcemia,Thyroidectomy,Thyroid disease,parathyroid

## Abstract

**Background:**

Early Hypocalcemia is the most frequent complication after thyroid surgery. Several studies have tried to identify factors (patient caracteristics or surgical technique variations) affecting hypocalcemia following thyroid surgery. This studiy evaluates the role of several factors in postoperative hypocalcemia development.

**Methods:**

A retrospective study conducted on 2108 patients that underwent thyroid surgery in a single center (1669 women and 439 men). Postoperative early hypocalcemia was defined as serum calcium levels lower than 8,0 mg/dl measured 24 h after surgery. Following factors were evaluated in the study: sex, age, glandular hyperfunction, preoperative diagnosis, preoperative serum calcium levels, preoperative serum PTH levels, type of surgery performed (total thyroidectomy vs. lobectomy); minimally invasive video assisted thyroidectomy (MIVAT); number of parathyroid preserved in situ, postoperative serum calcium levels, changes in perioperative calcium levels (difference between preoperative values ​​and postoperative calcium levels), presence of carcinoma in the surgical specimen, presence of thyroiditis based on histopatology reports.

**Results:**

Among evaluated factors only gender and surgical procedure revealed to be significantly correlated to early hypocalcemia development. In fact female patients experienced postoperative hypocalcemia in 42% (701/1669) of cases, which was signicantly higher than the 21.4% (94/439) identified in men. We also noticed a greater hypocalcemia incidence in patient undergoing total thyroidectomy (38.8%) than in patient undergoing lobectomy group (13.8%). Early hypocalcemia development didn’t appear to be related to preoperative serum calcium levels but it showed a statistically significant correlation with perioperative serum calcium level drop.

**Conclusion:**

This findings suggest that sex (female gender is a strong risk factor),surgical procedure and perioperative changes in serum calcium are the only factors (among all variables examined) that influence early hypocalcemia development.

## Background

Thyroyd surgery (total or near total thyroidectomy) can lead to serious complications, including transient or permanent cordal palsy or severe bleeding. However, hypocalcemia is the most frequent complication after thyroid surgery [1–3].

Transient hypocalcemia frequently complicates post-operative care of patients who have undergone thyroid surgery.

Post-thyroidectomy hypocalcemia arises because of parathyroid removal, devascularization and damage which induce a state of transient (or permanent) hypoparathyroidism.

Additional mechanisms, such as vitamin D deficiency, an acute increase in calcitonin serum levels (because of gland handling during surgery) or an “hungry bone syndrome” are believed to contribute to this process [4–8]. Etiological considerations include post-operative alkalosis-induced hypocalcemia resulting from hyperventilation triggered by postoperative pain, and diluition hypocalcemia [9].

Even though the perfect knowledge of thyroidal anatomy regarding the embriological origin of parathyroid glands is the most concrete element to decrease incidence of post-operative hypocalcemia [10].

Although the rate of of hypocalcemia has decrease as parathyroid preserving techniques have developed, the rates of transient hypocalcemia still range between 6.9 and 49.0% of patient undergone thyroid surgery [11–16].

Surgeon’s ability to predict the onset of post-thyroidectomy hypocalcemia is very important for post-operative management. Early detection of any risk of developing hypocalcemia will reduce the hospital stay lenght and eliminate unnecessary laboratory examinations.

When hypocalcemia is predicted, treatment with profilactic calcium and vitamin D supplements can prevent the development of hypocalcemia symptoms and premature discharge of patients. Several studies tried to identify risk factors related to early hypocalcemia (EH) development after thyroid surgery, with different results.

Post-operative hypoparathyroidism remains a clinical challenge for thyroid surgeons because of its frequency and the limited number of established preoperative predictors [4].

In this study, factors related to the patient as well as surgical methods have been analyzed to find a significant correlations between them and EH development.

## Methods

We have retrospectively analyzed the data of 2108 patients undergoing thyroid surgery at the Unit of General Surgery of the Parma University Hospital between January 2004 and June 2016. All surgeries were performed by the same surgical team experienced in endocrine surgery. All patients undergoing thyroid surgery were included regardless of the surgical indication for malignant or benign pathology.

We excluded patients undergoing thyroid surgery during the considered period whose postoperative calcium data were incomplete, patients with concomitant primary hyperparathyroidism, patients undergoing thyreoidectomy associated with neck dissection.

For each patient involved in the study, the following variables were analyzed.

Preoperative variables: Sex (further subdividing the female population in pre- and postmenopausal groups considering the age of 50 years as the limit between the two), Age (divided into 4 groups: age < 40, 40 < age < 50, 50 < age < 60 and age > 60 years), glandular hyperfunction, preoperative diagnosis (Benign pathology vs. malignant or suspected for malignancy disease), preoperative serum calcium levels, preoperative serum PTH levels (datas present for 813 patients), comorbidities (diabetes).

Surgical procedure characteristics: Type of surgery performed (total /near total thyroidectomy vs. lobectomy); traditional cervicotomic procedure vs. minimally invasive video assisted thyroidectomy (MIVAT); surgical approach to benign pathology vs approach for malignant pathology, number of parathyroid glands visualized by the surgeon during surgery (reported in 1202 cases).

Postoperative variables: Postoperative serum calcium levels (24 h after surgery), variations in perioperative calcium levels (difference between preoperative values and postoperative calcium levels), definitive histological examination report, presence of thyroiditis based on histopatology reports.

We have considered the presence of Early Hypocalcemia for calcium serum levels lower than 8.0 mg / dl measured 24 h after surgery.

Depending on the Early Hypocalcemia evidence, patients were divided into two groups and were compared considering the analyzed variables, attempting to highlight any statistically significant correlation between individual variables and the Early Hypocalcemia development.

Statistical data analysis was performed using SPSS (version 20.0; SPSS Inc. Chicago, IL, USA). To compare parametric variables, the Pearson chi-square test or Fisher’s exact test was used. To compare non-parametric variables, Student T test or Mann-Whitney test U was used. Level of statistical significance was determined at *p* < 0.05.

## Results

We analyzed datas about a total number of 2108 patients that underwent thyroid surgery in the study period. There were1669 women (79.2%) and 439 men (20.8%) with a mean age of 54.65 years (range, 15–87). Patients’ demographics, operative details, histological findings and postoperative events are reported in Table 1.

**Table 1:** Studied population characteristics

Variable	Total n. Of patients (2108)
Sex	
Female	1669 (79.2%)
Male	439 (20.8%)
Age (y)	
Mean ± sd	54.6 (± 13,85)
Range	15–87
Age groups	
< 40	309 (14.7%)
40–50[	439 (20.8%)
50–60[	547 (26%)
> 60	812 (38.5%)
Post-menopausal age (tot.1669)	
Yes	1054 (63.2%)
No	615 (36.8%)
Preoperative diagnosis	
Malignant	824 (39.1%)
Benign	1284 (60.9%)
Hyperthyroidism	
Yes	382 (18.1%)
No	1726 (81.9%)
Thyroiditis	
Yes	727 (34.5%)
No	1381 (65.5%)
Diabetes	
Yes	181 (8.6%)
No	1927 (91.4%)
Hypertension	
Yes	601 (28.5%)
No	1507 (71.5%)
Definitive histological diagnosis (tot. 1914)	
Malignant	667 (34.8%)
Benign	1247 (65.2%)
Surgical procedure	
Total thyroidectomy	2014 (95.5%)
Lobectomy	94 (0.5%)
Total number of parathyroid glands identified during operation (tot. 1202)	
0	16 (1.3%)
1	68 (5.7%)
2	389 (32.4%)
3	656 (54.6%)
4	73 (6.1%)
Calcium drop from preoperative to postoperative (24 h)	
Mean ± sd	1.203 (± 0.41) mg/dl
Postoperative early hypocalcemia (ca < 8.0 mg /dl)	
Yes	795 (37.7%)
No	1313 (62.3%)

We divided all studied patients in age groups: 309 (14.7%) patients younger than 40 years, 439 patients (20.8%) aged between 40 and 50 years, 547 patients (26.0%) aged between 50 and 60 years and 812 patients (38.5%) older than 60 years.

Women in postmenopausal age were 1054 (63.2%), the other 615 female patients (36.8%) were younger than 50 years when the surgical procedure was performed.

Considering Preoperative diagnosis we divided patients in two groups: 1284 patients (60.9%) underwent surgery for benign patology and 824 patients (39.1%) underwent surgery for malignant patology or suspected for malignancy. Thyroid Hyperfunction was present in 382 patients (18.1%). Thyroiditis was found in histopatological report of 727 patients (34.5%) the other 1381 patients (65.5%) didn’t show signs of glandular cronic inflammation.

Based on final histopatological reports (datas were present in 1914 cases) we found the following results: 1247 patients were diagnosed of benign patology (65.2%) while 667 patients have been found to be affected by malignant patology (34.8%). By analyzing the surgical procedure performed we found out that total thyroidectomy was performed in 2014 cases (95.5%) and lobectomy in 94 cases (4.5%).

We considered also the number of identified parathyroid glands during surgery (datas were present in 1202 cases): 0 glands identified in 16 procedures (1.3%), 1 gland identified in 68 procedures (5.7%), 2 glands identified in 389 procedures (32.4%), 3 glands identified in 656 cases (54.6%) and 4 glands in 73 procedures (6.1%).

Taking in consideration preoperative and postoperative serum calcium levels we found that there was a mean perioperative calcemia decrease of 1.203 (± 0.41) mg / dl.

Among patient comorbidities diabetes was present in 181 cases (8.6%) and a medical history of arterial hypertension has been described in 601 cases (28.5%).

Patients involved in the study were divided into two groups according to Early Hypocalcemia (EH) (postoperative serum calcium levels lower than 8 mg / dL) detection.

Serum calcium levels lower than 8.0 mg / dl 24 h after surgery were observed in 795 patients out of the 2108 examined (37.7%), thus forming the following 2 groups: Group N (normocalcemia) including 1313 out of 2108 patients (62.3%) and Group EH (hypocalcemia) including 795 out of 2108 patients (37.7%).

By analyzing patients mean age, the group of patients that developed EH shows a mean age of 55.03 (± 13.87) years and in the N group the mean age is 54.01 (± 13.47) years.

There was no statistically significant difference between the two groups.

Patients of both groups were then further subdivided into four age groups: patients under 40 years (*n* = 309) showed a development of Early Hypocalcemia in 36.9% (114 out of 309) of cases. Patients aged 40 to 50 years (*n* = 439) developed EH in 43.3% (190 out of 439) of cases; those patients aged between 50 and 60 years (*n* = 547) developed EH in 38.6% (211 out of 547) cases and among the over 60 years old patients (*n* = 821) the incidence of EH was 34.5% (280 out of 812) cases.

There were no statistically significant differences between the different age groups, confirming the absence of correlation between patient age in the EH development process.

Analyzing male and female population within the study group there was a statistically significant difference (*p* < 0.0001) between men and women regard EH development.

Among men (*n* = 439) included in the database, 21.4% (94 out of 439) developed EH, while women (*N* = 1669) showed EH in 42% of cases (701 out of 1669). Female gender therefore appears to be a predisposing factor for the EH development.

The female population was subsequently divided on pre and post-menopausal age groups. The premenopausal women group (615 out of 1669) presented EH in 45.2% of cases, while the postmenopausal women group (1054 out of 1669)in 40.1% of cases. There was no statistically significant difference between the two groups.

Female sex therefore appears to favor EH development, but there is no statistically significant difference between pre and post-menopausal patients.

There are no statistically significant reports on the occurrence of EH considering preoperative diagnosis, we identified two gropus: benign pathology (Thyr 2, toxic pathology or multinodular goiter) and malignant pathology or suspect for malignancy (Thyr 3, Thyr 4, Thyr 5 and Thyr 6 following the Bethesda 2010 thyroid nodule classification based on citological examination by needle aspiration).

In the group including patients undergoing thyroid surgery for benign pathology (1284), EH was found in 38.5% of cases (494 out of 1284). Patients that underwent surgery for malignant thyroid disease (824) showed EH development in 36.5% (301 out of 824) cases. (*P* = 0.382).

Correlation between preoperative PTH serum levels (data present in 813 cases) and the onset of EH was observed. In the EH group the mean PTH value was 59.03 (± 24.336) pg / ml, in normocalcemic group mean PTH serum level was 56.78 (± 21.195) pg / ml. There was no statistically significant difference between the two groups (*p* = 0.367). Preoperative PTH serum level therefore appears to have no influence on the EH development.

Preoperative mean serum calcium level in patients in the EH group was 9.172 (± 0.38) mg / dL, not highlighting a statistically significant difference with N group patients where preoperative mean serum calcium level was 9, 35 (± 0.43) mg/dl.

We noticed a statistically significant correlation between the variation of perioperative serum calcium level (difference between preoperative calcium and postoperative calcium) and EH development. In the EH group mean decrease in serum calcium value registered was 1.67 (± 0.49) mg / dl, in the N group the mean calcium level drop was 0.920 (± 0.47) mg / dl.

EH development does not therefore appear to be correlated with preoperative serum calcium level, but the breadth of the preoperative serum calcium decrease (*p* < 0.001) seems to play a crucial role.

Correlation between surgery performed (total thyroidectomy or monolateral lobectomy) and EH development was analyzed.

Patients undergoing total thyroidectomy (*n* = 2014) showed EH development in 38.8% (782 of 2014) cases, showing a statistically significant difference (p < 0.001) compared to the group including patients who underwent monolateral lobectomy (94 pts) which showed EH in 13.8% of cases (13 out of 94).

We also tried to analyze the correlation between EH development and thyroid hyperfunction (we considered patients undergoing surgery due to Graves’s disease or multinodular toxic goitre) but no statistically significant differences showed up. Among hyperthyroid patients (382), 38.8% (*n* = 148) showed EH development, among euthyroid patients this percentage reached 37.5% (647 out of 1726). Thyroid hyperfunction does not appear to affect the EH onset.

Correlation between final histopathological examination report (present in 1914 cases) and onset of EH was also observed.

Patients diagnosed with thyroid cancer with final histological examination (*n* = 667) showed EH in 40.5% (270 out of 667) cases; patients affected by benign pathology (*n* = 1247) developed EH in 36.7% (458 out of 1247) cases. Data analysis evidenced that there is no statistically significant correlation between the two groups (*p* = 0.114). In our study malignant thyroid disease does not therefore seem to affect the EH onset and surgery for malignant thyroid disease does not seem to be characterized by a higher incidence of EH.

We compared the number of parathyroid glands identified during surgery (data present in 1202 cases) and the occurrence of EH.

Evidence of EH was found in 38.4% (28 out of 73) of cases when 4 parathyroid glands were described, in 33.7% (221 out of 656) of cases when 3 glands were described, in 32.1% (125 Out of 389) of cases when 2 glands were identified by the surgeon and 20.6% (14 out of 68) of cases in wich a single parathyroid gland was shown intact. Among the 16 surgical reports that didn’t describe parathyroid glands, EH was recorded in 2 single cases (12.5%).

Thus a greater incidence of EH appeared when the number of parathyroid glands displayed during surgery increased, but this difference did not appear to be statistically significant (*p* = 0.63).

We tried to identify a possible effect of thyroiditis (diagnosed during the histopatological examination) on the EH development. Patients affected by thyroiditis (*n* = 727) showed EH development in 37.4% (272 out of 727) of cases, the other group, including patients not affected by thyroiditis showed EH development in 37.9% (523 out of 1381) of cases.

There was no statistically significant difference between the two groups (*p* = 0.837).

Within the studied population, 181 patients were affected by Diabetes (type 1 and type 2); we analyzed correlation between diabetic disease and EH onset. Patients with diabetes developed EH in 34.8% of cases (63 out of 181), non-diabetic patients showed EH in 38% of cases (732 out of 1927). Thus, the presence of diabetes does not seem to affect EH appearance, there is no statistically significant difference between the two groups (*p* = 0.399).

## Discussion

Hypocalcemia is a common complication after thyroid surgery. It usually occurs in first days after surgery and it can be symptomatic or asymptomatic. The frequency of transient hypoparathyroidism after thyroid surgery is between 6.9 and 49% [11–16].

The mechanism of hypocalcemia after thyroidectomy is not precisely disclosed, although is accepted to be multifactorial; factors like surgical techique, parathyroid iatrogenic damage (injury, edema, infarction, ischemia), extent of thyroidectomy, hyperthyroidism, malignancy, patient gender, perioperative serum calcium drop, presence of thyroiditis, diabetes, number of identified parathyroid gland during surgery can be considered as etiological factors [2, 17, 18].

Most studies underline the significance of expertise and surgeon’s experience.

According to Literature, in the present study we noticed that 37.7% of patients that underwent tyroid surgery in our Unit developed early hypocalcemia (EH), in fact we found serum calcium levels lower than 8.0 mg/dl 24 h after surgery in 795 patients on 2108. Because of this incidence we tried to find out patient caracteristics, disease related factors or surgical procedures that can influence the EH developmentt [11].

In literature, contrary opinions have been asserted about correlation between development of postoperative hypocalcemia and patient age. Some studies, found transient hypocalcemia to be associated with advanced age, whereas others reported an association with younger age. A systematic review performed by Edafe et al. Observed no significant difference in mean age between patients who had transient hypocalcemia and those who did not [18]. The present study also found no significant intergroup difference with regard to patient age. We also divided patients in four age groups (ved. Table 2) but no significant difference have been noticed between groups.

**Table 2:** Studied population related to hypocalcemia

Variable	Hypocalcemia (*n* = 795)	Normocalcemia (*n* = 1313)	*P* value
Sex			
female	701 (42%)	968 (58%)	*P < 0.0001*
male	94 (21.4%)	345 (78,6%)	
Age (y)			
mean ± sd	55.03 (±13,87)	54.01 (±13.47)	N.S.
range			
Age groups			
< 40	114 (36.9%)	195 (63.1%)	
40–50[	190 (43.3%)	249 (56.7%)	
50–60[	211 (38.6%)	336 (61.4%)	N.S.
> 60	280 (34.5%)	532 (65.5%)	
Post-menopausal age (tot.1669)			
yes	40.1%	59.9%	N.S.
no	45.2%	54.8%	
Preoperative diagnosis			
malignant	301 (36.5%)	523 (63.5%)	N.S.
benign	494 (38.5%)	790 (61.5%)	
Hyperthyroidism			
yes	148 (38.8%)	234 (61.2%)	N.S.
no	647 (37.5%)	1115 (62.5%)	
Thyroiditis			
yes	272 (37.4%)	455 (62.6%)	N.S.
no	523 (37.9%)	858 (62.1%)	
Diabetes			
yes	63 (34.8%)	118 (65.2%)	N.S.
no	732 (38%)	1195 (62%)	
Hypertension			
yes	181 (31.3%)	420 (68.7%)	*P < 0.001*
no	607 (40.3%)	900 (59.7%)	
Definitive histological diagnosis (tot. 1914)			
malignant	270 (40.5%)	397 (59.5%)	N.S.
benign	458 (36.7%)	789 (63.7%)	
Surgical procedure			
total thyroidectomy	782 (38.8%)	1232 (61.2%)	*P < 0.001*
lobectomy	13 (13.8%)	81 (86.2%)	
Total number of parathyroid glands identified during operation (tot. 1202)			
0	2 (12.5%)		
1	14 (20.6%)		N.S.
2	125 (32.1%)		
3	221 (33.7%)		
4	28 (38.4%)		
Preoperative calcium levels (mg/dl)			
mean ± sd	9.172 (±0.38)	9.35 (±0.43)	N.S.
Preoperative pth (pg/ml)			
mean ± sd (tot. 813)	59.03 (±24.336)	56.78 (±21.195)	N.S.
Calcium drop from preoperative to postoperative (24 h)			
mean ± sd	1.67 (±0.49)	0.920 (±0.47)	*P < 0.001*

In most studies women were found to have significant higher rates of hypocalcemia [11, 13, 18, 19] whereas other studies showed that gender has no significant effect on the incidence of hypocalcemia [11, 19–22].

According to literature we identified sex as significant risk factor for hypocalcemia, in fact female seemed to be more prone to develop this complication. In fact in our study female patients experienced EH in 42% (701/1669) of cases, which was significantly greater than the 21.4% (94/439) incidence detected in men (*p* < 0.001). There was no significant difference in rates of EH between premenopausal women and postmenopausal women, as confirmed by other studies [4].

Many studies tried to find an explanation to female predisposition to post-thyroidectomy hypocalcemia, but the specific mechanisms undelying this gender difference can only be assumed. The gender disparity may be related to effects of sex steroids on PTH secretion, genetic variation among cell-signaling pathways or anatomic differences that can cause more frequent iatrogenic damages because of a more diminutive operative field [4].

Some studies identified low preoperative level of serum calcium as a risk factor for the development of transient hypocalcemia [11, 19, 23–25]. In our study no difference has been identified between mean preoperative serum calcium level in EH group and in normocalcemic group. There was a significant difference in serum calcium level drop; mean perioperative variation in serum calcium levels (difference btween preoperative level and 24 h postoperative level) was significantly higher in patients that developed early hypocalcemia (*p* < 0.001). These findings clearly show that preoperative level of serum calcium has no influence on EH development, but perioperative level variation plays a decisive role in this process. This mechanism is confirmed by other studies in which a larger decrease in post-operative calcium from preoperativ levels was associated with transient hypocalcemia [19, 24, 26–28].

In literature hyperthyroidism is described as a risk factor for EH development; it is unclear why thyrotoxic thyroidectomies have an increased rate of hypocalcemia; however, it is perhaps unsurprising as the thyroid gland in thyrotoxicosis tends to be larger than normal and very highly vascularised leading to a more challenging operation [1, 29]. In our study thyroid hyperfuntion didn’t appear as a significant factor in EH development (Table 2) as confirmed by other studies in literature [9, 30].

Few articles in literature investigated effect diabetes has on hypocalcemia following thyroidectomy. Al-Dhahiri et al. Prospectively explored factors affecting recovery of parathyroid function after thyroidectomy and found diabetes mellitus to be a statistically significant factor.[1, 30]The mechanism by which diabetes cause this effect is unclear; however, it is hypothesised that the small vessel disease and the impact on angiogenesis may leave the parathyroids more vulnerable to hypoxia in these patients. This hypotesis is not confirmed by our study,no significant difference (*p* = 0.399) was found between diabetic patients and patients not affected by diabetes regarding EH development.

The surgical technique and the extent of thyroidectomy are related to parathyroid injury, edema, infarction, ischemia or incidental parathyroidectomy [2, 11, 18]. Dissection carried around the parathyroid glands and efforts to isolate RLN in this region can lead to venous congestion and edema. In addition, ligating of thyroid veins is among the cause of venous stasis. Venous stasis and edema slow down parathyroid function and may cause a temporary hypoparathyroidism [17]. As confirmed by our study, the incidence of hypocalcemia is much lower among patients that underwent lobectomy (13,8%) than in total thyroidectomy patient group (38.8%).

Some authors described thyroidectomy for carcinoma as a higher risk operation because in case of malignant pathology posterior capsule is radically removed with the gland and this is the reason why parathyroid glands are at higher risk of injuryas the risk of nerve injury [17, 31–34]. In our study, as it has been described also in other studies [16], EH developed in 36.5% of patients with preoperative malignant or suspected malignant (Thyr 3, Thyr 4, Thyr 5) diagnosis, and in 38,5% of patients that underwent surgery for benign pathology. Surgery for malignant pathology was not found as a significant factor for the development of EH.

The importance of systemic identification of all 4 parathyroid glands during thyroid surgery is one of the most controversial factors debated in the literature. Some authors recommend routinary physical identification and preservation of as many of parathyroid glands as possible [35]. Other series questioned this strategy [18, 36–39]. Among our patients we noticed an increasing rate of EH when a higher number of parathyroid gland have been identified during surgery, but statistical analysis didn’t show significant results (*p* = 0.63). To avoid potential injury to the parathyroid glands, every surgeon must be thoroughly aware of their anatomic complexity that contributes to difficulty of identification and possible injury. Strict adherence to capsular dissection represent the optimum method for safe preservation of parathyroid glands without necessitating their systemic identification. Distal ligation of all terminal branches of the superior and inferior thyroid arteries, close to the thyroid capsule, enables reliable separation of all tissues carrying parathyroid gland away from the thyroid surface. Continued dissection in this tissue, with the aim to identify all parathyroid glands may increase the risk of their mechanical injury or devascularization.

## Conclusion

This findings suggest that sex (female gender is a strong risk factor), surgical procedure and perioperative changes in serum calcium are the only factors (among all variables examined) that influence early hypocalcemia development.

All the risk factors detected in our study appear to be very common and not editable before nor during or after surgical procedure. This is the reason why in our unit we are used to suggest prophylaxis against symptomatic hypocalcemia (Carbonate calcium 1 g and Vitamin D 0,50 mcg per os twice a day for seven days) to every patient who underwent thyroid surgery. In our experience, therefore, prophylaxis with calcium and Vit. D (4 euros/patient) during hospitalization and after patient discharge was found to be beneficial both in terms of clinical outcome and in terms of health costs. Since when we started this prophylaxis we noticed a decreased length of stay and minimization of re-entry.

## Abbreviations

EH: Early hypocalcemia
MIVAT: Miniinvasive Videoassisted thyroidectomy
N: Normocalcemia
